# The importance of recovering body awareness in post-stroke rehabilitation: insights from clinical case reports

**DOI:** 10.3389/fneur.2024.1467181

**Published:** 2024-12-12

**Authors:** Davide Cardile, Viviana Lo Buono, Francesco Corallo, Simona Cammaroto, Caterina Formica, Angelo Quartarone, Rocco Salvatore Calabrò

**Affiliations:** IRCCS Centro Neurolesi Bonino-Pulejo, Messina, Italy

**Keywords:** body awareness, body image, neurorehabilitation, stroke, cognition, mood

## Abstract

**Introduction:**

Body awareness (BA) is the process of gaining sensory awareness based on the physiological states and actions of the body. It is influenced by an individual’s attitudes, perceptions, beliefs, and experiences within the social and cultural contexts. Following a stroke, impairments in BA are thought to be widespread and could have a significant impact on recovery results. Regaining body awareness, however, is often neglected in the neurorehabilitation field. This study aimed to assess body image perception in two stroke patients and the potential effect of motor and cognitive rehabilitative treatments on possible improvement of BA.

**Methods:**

Patients were evaluated through a multidimensional neuropsychological assessment before and after a 3-month motor and cognitive rehabilitative training. Sessions were scheduled 6 times per week with a total duration of 3 h per session.

**Results:**

After the neurorehabilitative treatment, both patients showed an improvement in BA, cognition, mood, and motor skills. Differences emerged related to the progression and improvement of their respective performances.

**Discussion:**

The causes of these differences could include the following: different brain areas affected, the ischemic or hemorrhagic nature of the stroke, age, and sex. Further research is needed to better understand the differences and similarities in the correlations between deficit and lesional sites. Structured and early multidisciplinary intervention can certainly guarantee a better functional recovery for patients after a stroke. However, in this study we show how complementary assessment methods (such as human figure drawing) may be highly informative in choosing treatment modalities and verifying rehabilitation outcomes.

## 1 Introduction

Body awareness (BA) is closely related to proprioception and is the sense that allows us to perceive the position, movement, and actions of our body parts, even without relying on visual input (1, 2). BA is considered to be an interactive process that includes awareness of the physiological states of the body, processes including pain and emotion, and activities, such as movement. Although this construct is predominantly thought of as related to the physical area, it is shaped by individuals’ attitudes, perceptions, beliefs, and social/cultural context experiences (3, 4). It involves receptors in muscles, tendons, and joints that send signals to the brain, allowing one to sense one’s own position of body in space (5). BA is a basic dimension in the concept of the body (6) and it develops from body schema and body image (7). Body image refers to the mental representation of one’s own body, arising from all sources of sensory and cognitive information, whereas body schema describes the unconscious use of sensory information by the motor system to maintain body posture and produce accurate movements. BA is the conceptualization of body image, and its conceptual realization, which is predicated on our perception of our experiences of bodies and our assessment of the data we gather from various sensorial sources (8).

Indeed, BA can be seen as the transition from objective to subjective sensory physiology (9). It is the interface between physiological body perception (i.e., visual, tactile, olfactory, gustatory, auditory, or kinesthetic as well as visceral perception) and cognitive-affective processing in the nervous system (10). Therefore, for effective processing and integration of this sensory information, the sensory pathways from the body to the brain must be intact. BA also subtends a visual representation of the body that can be investigated through the graphic representation of one’s own body. This, combined with the integration of multisensory neural inputs, makes BA an essential aspect of embodied self-awareness (11–13). Adding to the complexity of this construct are the involvement of different anatomical sites and the presence of higher order cognitive processes that decode and modulate these sensory inputs (14). For this reason, among the most used assessment instruments in literature there are tests such as the Multidimensional Assessment of Interoceptive Awareness (MAIA), the Body Perception Questionnaire (BPQ), and the Body Awareness Questionnaire (BAQ), which multidimensionally evaluate BA assessing motor awareness/perception, and the level of consciousness related to one’s internal sensations (15–17).

Serrada et al. (18) in their longitudinal study sought to investigate whether and how BA changed over time in a sample of more than 100 stroke patients, and to do so, they considered both the administration of the MAIA with that of the body perception disturbance (BPD) (19). In doing so, they smartly combined proprioceptive and interoceptive BA measures. Interoception is the perception of internal states, such as hunger, thirst, heartbeat, and visceral sensations, and is crucial for maintaining homeostasis and emotional regulation (20, 21). On the other hand, proprioception refers to the sense of the position and movement of body parts in space, mediated by mechanoreceptors in muscles, tendons, and joints, and is essential for coordinated motor control and balance (5, 22). These two constructs can be affected concomitantly or independently when conditions that impair BA occur, including several neurological conditions such as traumatic or vascular brain injury (23). Numerous people experience impairments in sensation and perception after a stroke which interrupts the representation of the body that is held in the brain (24). Clinically BA can be presented with distorted perception or reduced awareness of parts of the body or an impaired ability to localize them, feelings of strangeness, and illusory feelings of movement (25). After stroke, BA alterations are often associated with syndromes arising because of parietal lobe lesions (26). BA alterations may arise after a lesion in the posterior parietal cortex including the medial area, i.e., the precuneus, and more specifically the antero-dorsal precuneus (27). However, the neuroanatomical basis influencing BA includes an integrated system of brain regions and functional networks and involves the thalamus, insula, and cerebellum (3). Contrary to the general idea that BA alterations are predominantly associated with neglect and right hemisphere damage, literature data do not provide evidence for such hemispheric lateralization (28). BA training is crucial in patients who have suffered a stroke because focusing on both physical and cognitive functions it can improve clinical status helping regain the sense of their body in space and enhances their ability to control movements. For these patients, being able to recognize the position and movement of their limb enhances proprioception and facilitates better control and coordination of movements, positively impacting on daily activities and overall mobility (29–31). By improving body awareness, patients are better able to relearn motor skills and adapt to new movement patterns, which is crucial for regaining independence in daily life activities after a stroke (32). The improvement in BA also enhances balance, spatial awareness, and orientation, reducing the risk of falls (33). In addition, emotional regulation, anxiety, frustration, and pain management resulting from post-stroke physical limitations improve, as the rehabilitation process causes patients to regain confidence about their abilities and movements (17, 34).

Several authors have shown that recovery of BA is possible if patients are treated with adequate rehabilitation interventions (35–37). The limited available literature shows that BA in stroke patients is rarely addressed through stimulation or rehabilitation of both motor and cognitive abilities. Additionally, treatment outcomes related to BA are often considered only from a “physical” perspective such as improvements in walking, balance, or the movement of specific body parts, rather than from a proprioceptive standpoint. This study aimed to assess body image perception in two stroke patients and the potential effect of motor and cognitive rehabilitation on possible improvement of BA.

## 2 Clinical cases

This study included two stroke patients with BA impairments who were assessed and admitted to a rehabilitative program within 3 months after stroke onset. After being verbally informed in the presence of caregivers and all professionals involved in the rehabilitation process, verbal and written consent was obtained from both patients to enter the study and to publish the results.

### 2.1 Patients

At baseline, patients underwent neurological and neuropsychological examinations that revealed alterations in BA with fluctuating interoceptive and/or proprioceptive impairments. Patients did not present agnosia and neglect measured by the behavioral inattention test, and psychiatric history. Both patients were enrolled for specific rehabilitation training once they were medically stable, and the diagnosis of stroke was confirmed through MR imaging.

**Figure 1:**
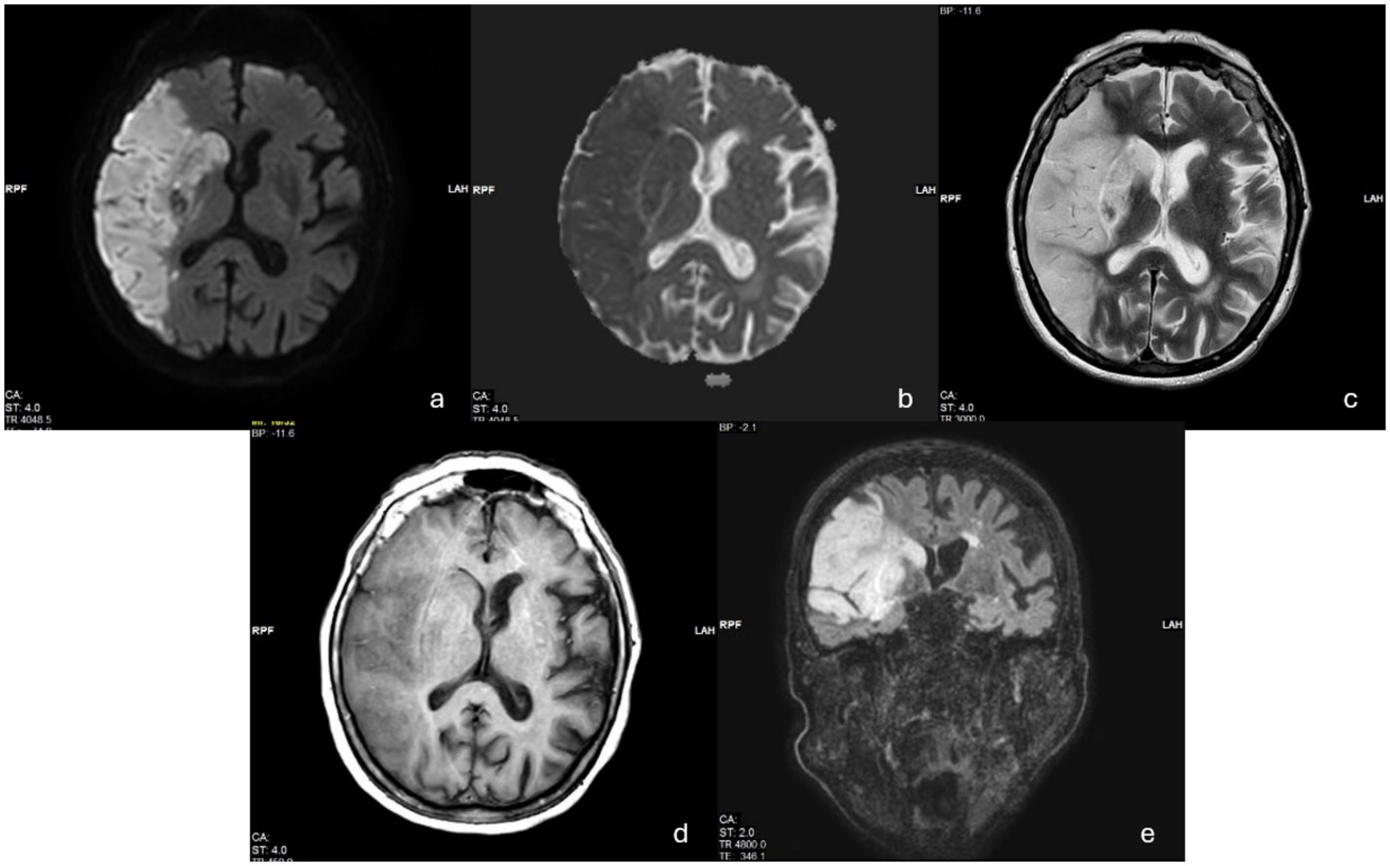
Diffusion images **(a,b)** show a signal restriction in the right middle cerebral artery territory with involvement of the frontal, parietal temporal, and ipsilateral capsular nucleus regions. The hyperintensity in T1 **(c)** shows that the ischemic event is in the late subacute phase, while the T1 image **(d)** shows the edematous condition and the reduced representa of the subarachnoid spaces as well as the imprint on the ipsilateral ventricle system and more so on the frontal horn. Coronal section **(e)** show the lesional areas.

**Figure 2:**
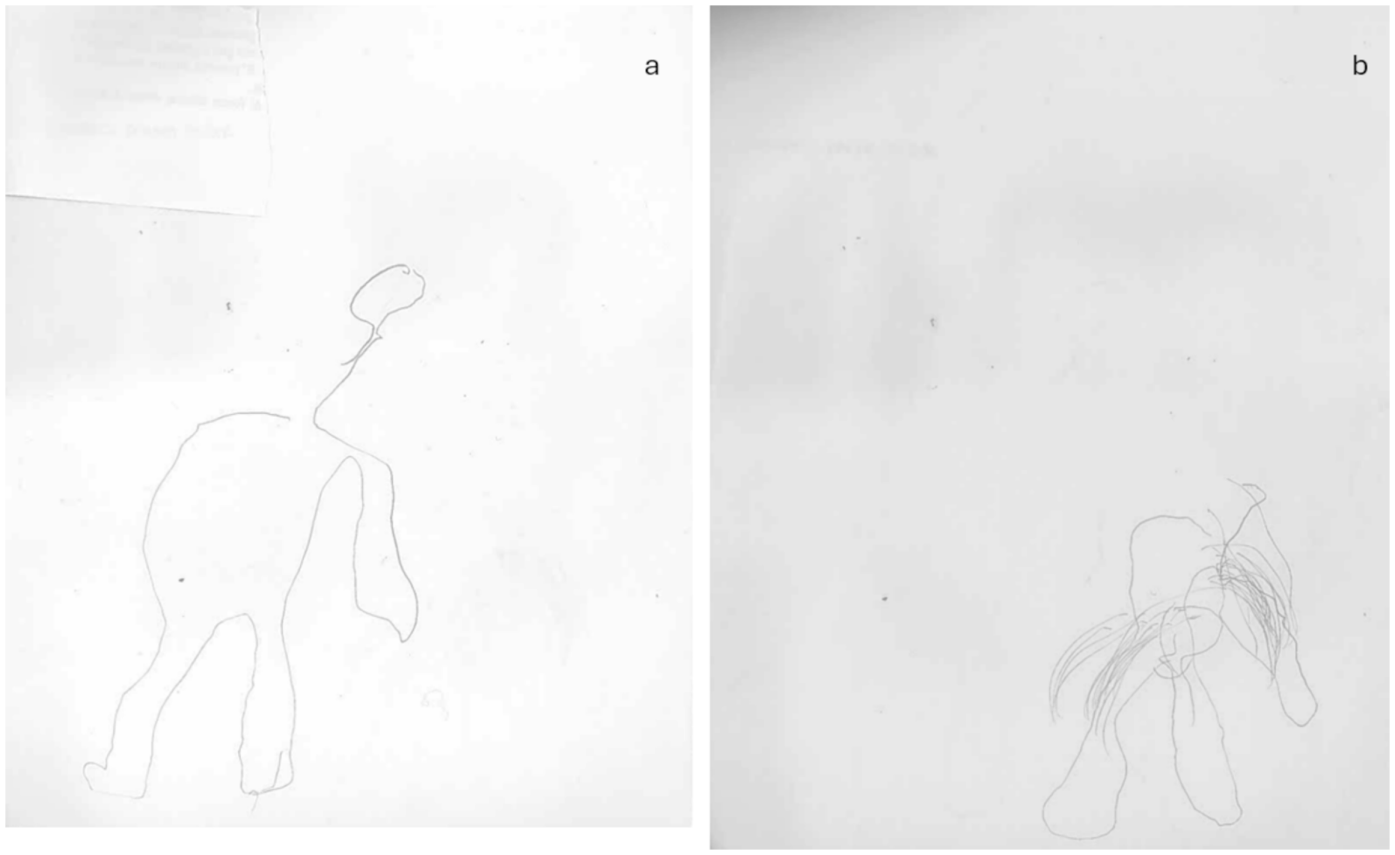
Graphical representation of the human figure of patient 1 at T0: **(A)** male representation; **(B)** female representation.

Case 1: A 65-year-old male right-handed, with obesity and hypertension, was affected by an ischemic stroke, occurring approximately 3 months before our observation (July 2021). Medical history was negative for other clinical events (i.e., previous stroke or heart diseases). At the neurological examination, the patient showed moderate upper left limb motor impairments, with muscle weakness, loss of dexterous movement, and sensory loss involving the tactile and proprioceptive modalities. No visual changes were recorded (NIHSS 13; Rankin Scale 3). The magnetic resonance image (MRI) showed a T2-weighted hyperintense lesion involving the right fronto-temporal–parietal area (Figure 1). The neuropsychological assessment showed a global cognitive decline and deficit in executive functions, visual–spatial ability, attention, and memory (Table 1). The patient also presented with severe anxiety and moderate depression disorders. The human figure drawing (HFD) showed alteration in BA. Indeed, the patient had a disturbance of the body schema characterized by a loss of ability to localize, recognize, and identify specific body parts to verbal commands. Both human figures represented (see Figures 2A,B) appeared incomplete in their corporal general characteristics. In particular, in Figure 2A the drawing was positioned at the left end of the sheet, and this could even be indicative of the presence of depressive feelings or an emotional state of introversion and withdrawal (38–40). The graphic stroke was weak and discontinuous; the basic elements that make up the human figure, such as the face (eyes, nose, mouth, and ears), hair, hands, and feet, were missing. The absence of the left arm seems to recall the patient’s left hemiplegia. In Figure 2B, no elements recall the features of a female human figure except for some features that evoke hair. The two lower limbs and the right upper limb were present, but the left upper limb was absent. The graphic stroke was weak and inaccurate.

**Table 1:** Neuropsychological evaluation at admission to neurorehabilitative training (T0) and after 3 months (T1).

	Patient 1			Patient 2		
TEST	(T0)	(T1)	RCI	(T0)	(T1)	RCI
MoCA	11	14	1.9	16	21	1.9
HAM-A	25	8	3.2	19	8	2.7
BDI-II	29	19	3.3	17	11	2.5
BIS	23	10	3.3	10	5	2.1
CIRS	3	3		2	1	
MAIA						
Noticing	20	35	1.9	30	45	3.9
Not distracting	70	67		80	80	
Not worrying	40	48	2.1	44	48	
Attention regulation	14	40	4.1	26	49	2.9
Emotional awareness	24	44	3.8	36	60	4.2
Self-regulation	20	25	1.9	25	45	3.7
Body listening	27	40	2.6	20	47	4.1
Trusting	20	47	3.9	40	67	4.1
FIM (total)	50	83	4.2	61	92	4.5
BBS	26	38	2.1	34	42	1.9
HFD (male)	6	10	1.9	11	13	
HFD (female)	6	8		10	14	1.9

*The Montreal Cognitive Assessment (MoCA), the Hamilton Anxiety Rating Scale (HAM-A), the Beck Depression Inventory—II (BDI-II), the Body Image Scale (BIS), the Clinical Insight Rating Scale (CIRS), the Multidimensional Assessment of Interoceptive Awareness (MAIA), the Functional Independence Measure (FIM), the Human Figure Drawing Test (HFD), and the Berg Balance Scale (BBS).

**Figure 3:**
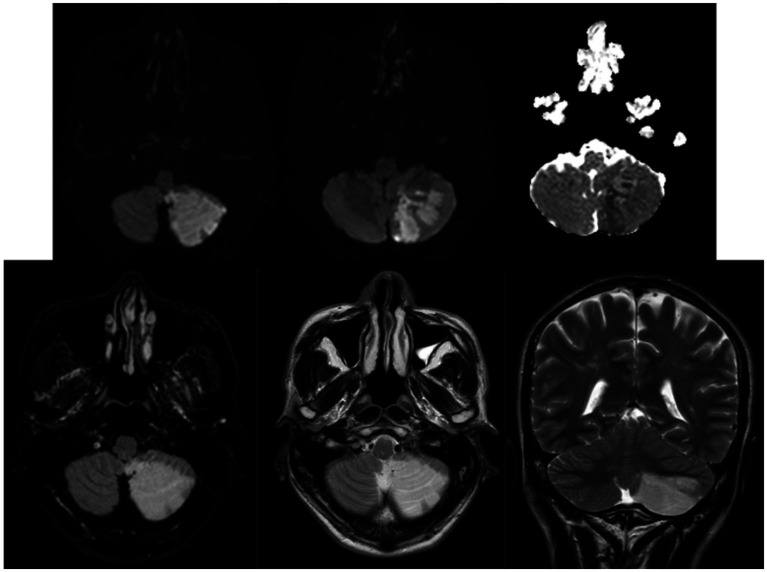
Case 2 MRI examination demonstrates the presence of an ischemic lesion in the left PICA area.

**Figure 4:**
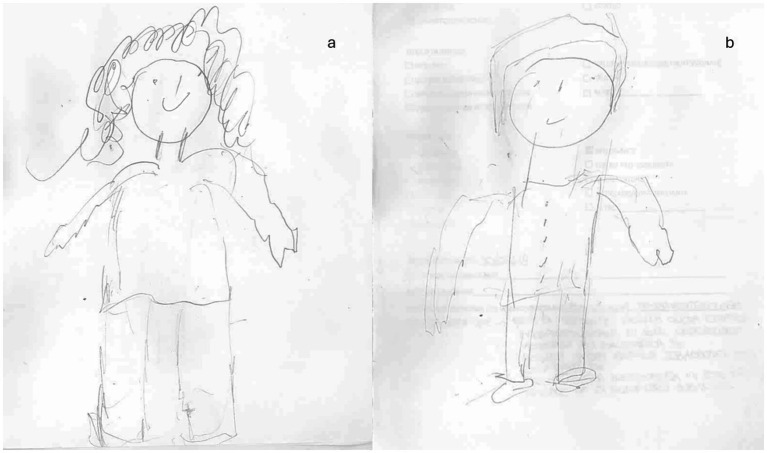
Graphical representation of the human figure of patient 2 at baseline T0: **(A)** female representation; **(B)** male representation.

Case 2: A 55-year-old right-handed woman suffered a hemorrhagic stroke that occurred less than 3 months before our assessment (March 2022). The lesion involved the left side of the posterior inferior cerebellar artery (PICA) area. At the neurological examination, she presented with a postural deficit, ataxia during gait, and limb incoordination with moderate left hypoesthesia (NIHSS 15; Rankin Scale 3) (Figure 3). The patient reported vertigo. The neuropsychological evaluation revealed a poor global cognitive performance, with deficits in visuospatial planning, executive functions, and memory domains. No visual alterations were recorded. In the HFD, alterations in BA emerged (Figures 4A,B). The female representation (Figure 4A) had large dimensions and was represented in the center of the sheet. Although several details on the face were present, the absence of feet may indicate a sign of instability due to a lack of balance. The left side had a less fluid trait, probably related to the continuous sensations of dizziness referred by the patient. The male figure was abnormal in dimension (Figure 4B). The fact that his head and neck were wider than his lower body in the drawing could on the one hand reflect her difficulties in balance walking, but also be indicative of poor spatial planning. In addition, this patient reported important alterations in body perception referring to abnormal sensations of the left side of her body.

### 2.2 Assessment and outcome measures

Given the importance of the early assessment of motor performance and cognitive functioning to develop an appropriate early rehabilitation program to maximize recovery, both patients were evaluated at admission to neurorehabilitation training (T0). After 3 months, a second evaluation was performed at the end of rehabilitative training (T1). To assess the actual significance of the improvements achieved by the patient (between T0 and T1) in all tests included in the neuropsychological assessment, we used the Reliable Change Index (RCI).

RCI is a standardized score used to determine whether a change in an individual (or group) performance is statistically significant based on the test–retest reliability of the measurement. RCI is calculated by dividing the change in the score of an individual by the standard error of the difference for the test being used. The cutoff value for statistical significance within the RCI is ≥1.96 (1.96 equates to the 95% confidence interval) (41).

The neuropsychological assessment included the administration of the following tests: the Montreal Cognitive Assessment (MoCA), the Beck Depression Inventory-II (BDI-II), the Hamilton Rating Scale for Anxiety (HAM-A), the Clinical Insight Rating Scale (CIRS), the Multidimensional Assessment of Interoceptive Awareness (MAIA), the Body Image Scale (BIS), the Human Figure Drawing Test (HFD), the Functional Independence Measure (FIM), and the Berg Balance Scale (BBS). The MoCA is a screening tool that evaluates several cognitive domains: attention and concentration, executive functions, memory, language, visuoconstructive skills, abstraction, computation, and orientation (42). The administration time is approximately 10 min and scores range from 0 to 30, with a score of 26 and above generally considered normal. BDI-II is a 21 multiple-choice item questionnaire used to measure the severity of depression in adults and adolescents at least 13 years old (43). HAM-A is a 14-item rating scale developed to measure the severity of anxiety symptoms. The test duration is short (10–15 min), and the total score goes from 0 to 56 allowing to discriminate between mild, moderate, and severe anxiety (44). CIRS is a standardized tool for evaluating awareness levels of cognitive and functional impairments and disease progression (45). MAIA is a tool that measures interoceptive awareness, the perception of sensation from inside the body including noticing, distracting, worrying, attention regulation, emotional awareness, self-regulation, body listening, and trusting (46). BIS is a 10-item Likert-scale questionnaire to assess body image concerns and changes over time (47). HFD is a test developed to evaluate the level of body awareness and that allows one to obtain a qualitative score about emotional status, personality traits, and the relationship between their body image perceived and self. Quantitative scoring was also performed according to other similar studies to compare pre-post performances (48). During the test, the patient is provided with a pencil and a blank sheet of paper, and he or she is required to draw a human figure. Subsequently, the patient must draw another human figure of the opposite sex of the first—scores obtainable range from 0 to 29 with lower scores indicating worse body perception (49). The Functional Independence Measure (FIM) scale was used to assess physical and cognitive disability (50). The scale includes 18 items, of which 13 items are physical domains and 5 items are cognition items. Each item is scored from 1 to 7 based on the level of independence, where 1 represents total dependence and 7 indicates complete independence. FIM total score can range from a minimum of 0 to a maximum of 126. The Berg Balance Scale (51) is a scale composed of 14 items of rapid administration (15 min) that aims to assess daily life movements such as the abilities to maintain and change positions, make movements with increasing speed, and perform tasks in unstable positions. Based on skill grades, each item is given a score from 0 to 4, where 0 is the minimum score and 4 is the maximum score. Summing the score obtained for all items gives a total score that will indicate the person’s state of mobilization: from 0 to 20, wheelchair; from 21 to 40, walks with assistance; and from 42 to 56, independent walking.

### 2.3 Rehabilitative program

Neurorehabilitative training was provided six times per week, and it included both motor and cognitive training. For both patients, motor training was performed by the same physiotherapist who is experienced and trained in the provision of physical therapy in patients with acquired brain injury. Motor training involved a series of exercises that targeted the learning of strategies to improve balance, coordination, flexibility, overall proprioceptive orientation, and gait training to improve mobility (52, 53). The sessions were aimed at sensorimotor skills relevant to basic daily functions, such as getting up from lying, sitting, rolling, and turning (54). Specifically, the following rehabilitation plan was established:

- Physical therapy with early mobilization of patients that included passive range-of-motion exercises to prevent joint stiffness and gradually progress to active-assisted and active exercises as the patients have gained strength.
- Proprioceptive training with weight-bearing exercises (standing, shifting weight from one leg to another) and balance exercises using a balance board and tandem walking.
- Strength and coordination exercises that required functional tasks such as reaching, grasping, and manipulating objects, and resistance training to improve muscle strength and endurance.

The cognitive training was delivered by an expert neuropsychologist who designed it as a combination of direct training of the impaired functions and metacognitive training to facilitate the development of compensatory strategies; it was based on cognitive and motivational techniques. The rehabilitative program was organized according to a predefined scheme. Attention training with exercises to improve focused, sustained, and divided attention; Memory exercises with tasks to enhance working memory, such as remembering and performing sequences of movements; executive function training including problem-solving tasks and planning activities that involve body movements; Motor imagery with the guided mental practice of movements to enhance motor planning and awareness.

Every day rehabilitative program was organized according to a predefined scheme: motor exercises (120 min) and cognitive exercises (60 min). The rehabilitation program lasted 12 weeks. Throughout the rehabilitation program, the difficulty of exercise progressively increased, ensuring a gradual advancement in both motor and cognitive abilities (Table 2). This structured approach remained consistent throughout the training period, with the complexity of tasks steadily rising over time.

**Table 2:** Rehabilitation program description with cognitive and motor tasks.

Rehabilitation program	Rehabilitative intervention	Session duration	Type of treatment
3 mo (July–October 21 for Case 1 March–June 22 for Case 2)	Motor training	6 weekly sessions of 120 min (72 total treatments)	Physical therapy—40 min: 20-min passive−/active-assisted mobilization of lower limbs and the trunk. 20-min trunk control exercises (sit-to-stand activities, training for postural changing). Proprioceptive training—40 min: 20-min static balance activities (standing with different variations in the base of support, tandem standing, and shifting the center of gravity using an oscillating platform). 20-min dynamic balance activities (standing up/sitting down with or without using hands, walking in tandem, lateral weight shifting, stationary stepping, walking with frequent stops and changes in direction, and postural variations). Strength and coordination exercises—40 min: 20-min sit-to-stand exercise and squats 20-min static–dynamic balance activities (walking while holding a ball, a plate, or a tower of glasses in the palm during stable balance conditions).
	Cognitive training	6 weekly sessions of 60 min (72 total treatments)	Reality-orientation therapy (ROT)—10 min: 5-min temporal/spatial orientation 5-min personal orientation (emotional/autobiographical/motivational training) Attention training—20 min: 5-min visual research/selective attention 5-min divided/alternating attention 5-min sustained attention Memory Training—15 min: 5-min visuospatial/verbal memory 10-min working memory (remembering/performing sequences of movements) Motor imagery—15 min: 5-min motor imagery 10-min executive planning/awareness

## 3 Results

After the neurorehabilitative treatments, both patients improved in BA, cognition, mood, and motor skills (Table 1).

**Figure 5:**
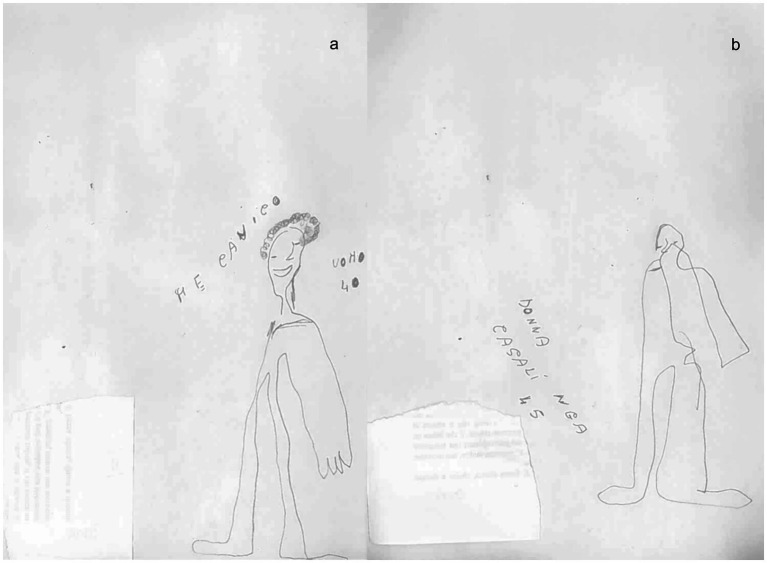
Graphical representation of the human figure of patient 1 after rehabilitative training T1: **(A)** male representation; **(B)** female representation.

In particular, at T1 concerning BA, the analysis of human figures (see Figures 5A,B) of patient 1 showed a better representation of all body elements with an overall increase in performance between 6.9 and 13.8%. Although in the drawing, it was still missing the left arm, it shows more harmonious proportions between the different parts of the body. The graphic stroke appears more careful and precise (shading of the hair). Cognitively, the score at MOCA increased at T1 (Table 1), while emotionally, the scores markedly decreased at both HAM-A (8) and BDI-II (19).

In addition to this improvement, also balance and autonomy improved, as revealed by scores recorded at the BBS and FIM. The MAIA test showed better performance (between 13 and 27% more), especially in the areas of noticing, attention regulation, emotional awareness, body listening, and trusting.

**Figure 6:**
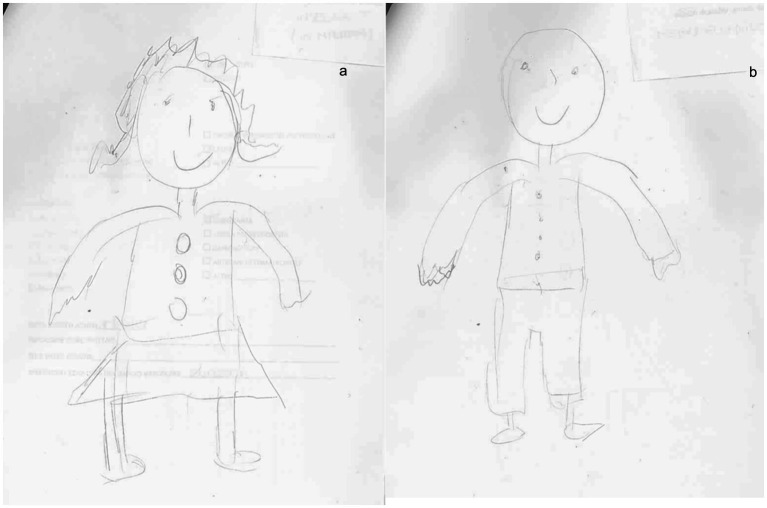
Graphical representation of the human figure of patient 2 at T1: **(A)** female representation; **(B)** male representation.

After neurorehabilitative training, patient 2 improved in BA with an overall increase in performance between 6.9 and 13.8%. At T1 (see Figures 6A,B), the dimensions of both figures were improved. The female figure was represented by more details concerning the male representation. This could indicate a clear sex identification of the patient. Although she started with higher scores on average, even in the second patient there was an increase in the score on the MoCA at T1. Moreover, there was a notable decrease in scores on both the HAM-A (8) and the BDI-II (11). Balance and autonomy scores improved, and the MAIA test revealed greater enhancements in the same areas as patient 1, with a performance between 23 and 27% better. In particular, also self-regulation showed an important increase (20%).

## 4 Discussion

This study sought to investigate body image perception in two stroke patients and the potential effect of motor and cognitive rehabilitative treatments in improving BA and functional deficits after stroke. Both patients involved in this study had similar issues related to BA with fluctuating interoceptive and/or proprioceptive impairments, although such alterations were caused by a completely different site of the lesion. The first patient presented a brain lesion in the right front-parietal–temporal area while the second one in the left side of the PICA. In line with other studies in literature, we found that motor and cognitive improvement was correlated with improvement in BA scores. Battesha et al. (55) and Sengar et al. (56) indeed treated stroke patients with treatments that considered both motor and cognitive aspects. In the former case (55), patients received physical rehabilitation in addition to maze control training. Specifically, patients were standing on a platform that had a motion, and they were asked to follow a set of targets through a maze. The participant moved a cursor to each target as it blinked and did not let the cursor touch a wall of the maze. On the other hand, in Sengar et al. (56) patients received dual-task balance training with postural (step forward, backward, and sideways) and cognitive tasks (remembering words and counting forward and backward by adding 3 to the digits). Both studies treated patients with middle cerebral artery stroke and found that combined (motor and cognitive) treatment improved physical awareness of limb or body part proprioception/position, balance, and autonomy. In this regard, Perfetti in his neurocognitive rehabilitation theory argues that the learning (or re-learning) of interaction patterns with their surroundings activates cognitive processes that produce sensory–motor recovery (57, 58). Salles et al. (59) relying on this theory, proposed an interesting cognitive therapeutic protocol that considered an individual’s motor, sensory, and cognitive characteristics using both kinesthetic and tactile tasks.

Although our results have been reflected by those in literature, to the best of our knowledge, this is the first study where the level of BA is not only assessed by motor and neuropsychological parameters but also by using HFD, a test that provides information about the patient’s representation and awareness of his/her body. The details in drawings, indeed, offer insights into BA and cognitive status (60, 61), with a particular focus on visuospatial and constructive impairments (62). In addition, drawing can serve as a qualitative tool to investigate also emotional status and mood disorders, it can be particularly suitable for assessing BA after a stroke. After the neurorehabilitation training, both patients presented a better graphical representation of themselves. Specifically, concerning HFD we observed more details and a better-defined trait in the human figure, especially about the figures of case 2 (Figure 6). In case 1, at T1, we observed little improvement in cognitive functions, and in details of HFD; however, the left upper limb remains unrepresented in all drawings (Figure 5). It is possible that the difficulty of hemiplegic upper limb recovery was related to a deficit in recognition of this part of the body in HFD. In addition to the improvement in balance and autonomy, the patient was more able to sustain and control attention to body sensations consciously. This would suggest that the rehabilitation training has produced cascading effects not only on the patient’s attention but also on the patient’s cognitive, emotional, and functional aspects. On the other hand, in case 2, we not only observed a greater improvement in cognitive domains and BA (Figure 6) but also a better ability to regulate anxiety and distress by paying mindful attention to bodily sensations. This led patient 2 to experience her body as more secure and trustworthy, which positively reinforced her attempts at attentional focus on body districts and sensations. Chiaramonte et al. (63) in one of their studies focused on patients’ characteristics and improvement to combined traditional rehabilitation exercises with goal-oriented proprioceptive exercises to improve not only motor performance but also representation of the body and its movements in space.

In case 1, lesions involved the parietal lobe and it is well known that this area has an important role in the function and processing of sensory information, understanding spatial orientation, and BA (64). In case 2 strokes hit cerebellar areas. Although BA deficits are most often associated with right parietal and temporo-parietal lesions, they may sometimes occur in association with lesions of the cerebellum and brainstem (65–67). Cerebellar damage frequently gives rise to a diverse range of cognitive dysfunctions, via the cerebrocerebellar loop; however, cerebellar networks are endowed with great plastic potential supporting a variety of learning mechanisms (68). This plasticity in the corticocerebellar system emphasizes the therapeutic potential of the cerebellar system for the enhancement of stroke recovery (69).

The ability to continuously observe, update, and provide input regarding one’s body’s position and movement in space is what makes BA so important. Accurate bodily information is crucial for the exact control of motions as it is the primary process utilized to integrate information for perception, decision-making, and action (24).

It is crucial to remember that the BA receives input from both external and internal senses. Gomez-Andres et al. (70) used auditory feedback-based rehabilitation via real-time provided to the patient supplementary information about his/her body position. While walking, alterations in frequency spectra of footstep sounds were induced via a system that selectively amplifies and equalizes the signal to generate distorted auditory feedback in the presence of an error. Their results show that feedback increased movement, coordination, and BA.

The limbic system provides affective and memory input related to body image, while the language and spatial portions of the parietal lobes in each hemisphere provide input related to semantic and lexical features. The ability of the person to maintain motor function and recuperate after a stroke is greatly aided by their intact BA, and this is what makes assessment and rehabilitation so important (71). However, after a stroke, many people report deficits in sensation and perception, which disrupts the representation of the brain of the body and significantly affects a person’s BA (72). Inaccurate perception of the body is caused by changed motor and sensory cortical processing, and it makes it more difficult to move with accuracy and control as it affects postural control, dynamic balance, coordination, and the person’s capacity to safely explore their immediate surroundings. It thus impacts one’s functional capacities, ability to carry out everyday tasks, and quality of life (73). Depending on the type of injury, rehabilitation can take a considerable amount of time (74, 75). Additionally, fluctuating or persistent alterations in neurocognitive performance make rehabilitation particularly challenging. As BA is often impaired in stroke patients and not always recovers spontaneously, neurorehabilitation is necessary to elicit and sustain it (76). Moreover, the length of rehabilitation and the destination of discharge are frequently hampered by decreased BA. Structured and early multidisciplinary intervention can certainly guarantee a better functional recovery for patients after a stroke. However, in this study we show how complementary assessment methods (such as HFD) can also be highly informative in choosing treatment modalities and verifying rehabilitation outcomes.

Unfortunately, we cannot draw important conclusions from our study (although the RCI was high for many items concerning BA and cognition outcomes), as it is based only on two case reports that have different etiology, and what is more, the lack of an adequate follow up prevents us to state to what extent the post-training effect lasts. However, BA deficit is poorly investigated in literature, and this overlooked problem may seriously affect post-stroke functional recovery and then deserve future investigation.

## 5 Conclusion

With this study, we showed the relationship between BA training and motor and neuropsychological rehabilitative outcomes and that the same type of treatment could be applied with positive results in patients with BA due to different brain lesions (i.e., fronto-temporo-parietal and cerebellar). Further studies should better investigate the role of the cerebellum in body image and cognitive functions, trying to better understand the neurophysiological connection with the main brain areas subtending BA. Moreover, more specific tests as well as the drawing test should be validated as qualitative tools to provide useful information about body image representation in patients with stroke and to monitor the progress of the motor and cognitive rehabilitative treatments.

## Data Availability

The datasets presented in this article are not readily available because of ethical and privacy restrictions. Requests to access the datasets should be directed to the corresponding author.
